# Intestinal parasite infections and associated risk factors among schoolchildren in Dolakha and Ramechhap districts, Nepal: a cross-sectional study

**DOI:** 10.1186/s13071-018-3105-0

**Published:** 2018-09-29

**Authors:** Akina Shrestha, Christian Schindler, Peter Odermatt, Jana Gerold, Séverine Erismann, Subodh Sharma, Rajendra Koju, Jürg Utzinger, Guéladio Cissé

**Affiliations:** 1 Swiss Tropical and Public Health Institute, P.O. Box, CH-4002 Basel, Switzerland; 2 University of Basel, P.O. Box, CH-4003 Basel, Switzerland; 3 Kathmandu University, School of Medical Sciences, P.O. Box 11008, Kathmandu, Nepal; 4 Kathmandu University, School of Science, Aquatic Ecology Centre, P.O. Box 6250, Dhulikhel, Nepal

**Keywords:** Helminths, Hygiene, Intestinal protozoa, Nepal, Sanitation, Water

## Abstract

**Background:**

Infections with soil-transmitted helminths and pathogenic intestinal protozoa pose a considerable public health burden, particularly in low- and middle-income countries, including Nepal. We assessed the extent of intestinal parasite infections among schoolchildren in two districts of Nepal and determined underlying risk factors.

**Methods:**

A cross-sectional survey was conducted between March and May 2015 in the districts of Dolakha and Ramechhap, Nepal. A total of 708 children, aged 8–16 years from 16 purposively selected schools, were enrolled. Each child provided a single stool sample that was subjected to a suite of copro-microscopic diagnoses for intestinal protozoa and helminths. Drinking water samples from different sources at schools (*n* = 29), community places (*n* = 43) and households (*n* = 562) were analysed for contamination with thermotolerant coliforms (TTC). A questionnaire was administered to determine individual- and household-level risk factors of intestinal parasite infections. Self-reported symptoms were assessed and a clinical examination was undertaken by a physician. Haemoglobin was measured and used as a proxy for anaemia. Mixed logistic regression models were applied to investigate associations.

**Results:**

The overall prevalence of intestinal parasite infections was 39.7%. *Trichuris trichiura* (30.9%), *Giardia intestinalis* (30.5%) and hookworm (30.2%) were the predominant intestinal parasite infections. Children from households lacking soap for handwashing were at higher odds of intestinal parasite infections than children who had soap [adjusted odds ratio (aOR) 1.81; 95% confidence interval (CI): 1.13–2.89; *P* = 0.01]. Children from households without freely roaming domestic animals showed lower odds of *G. intestinalis* compared to children from households with freely roaming animals (aOR 0.52; 95% CI: 0.33–0.83; *P* = 0.01). One out of three (31.0%) children suffered from fever and 22.4% had watery diarrhoea within a two-week recall period. Anaemia was diagnosed in 23.6% of the children. Water contamination with TTC showed no clear association with intestinal parasite infection.

**Conclusions:**

Intestinal parasites are common among schoolchildren in the two surveyed districts of Nepal. An important risk factor was lack of soap for handwashing. Our findings call for efforts to control intestinal parasite infection and emphasis should be placed on improvements in water, sanitation and hygiene interventions.

**Trial registration:**

ISRCTN17968589 (date assigned: 17 July 2015).

**Electronic supplementary material:**

The online version of this article (10.1186/s13071-018-3105-0) contains supplementary material, which is available to authorized users.

## Background

Intestinal parasite infections caused by soil-transmitted helminths (e.g. *Ascaris lumbricoides*, hookworm and *Trichuris trichiura*), and pathogenic intestinal protozoa (e.g. *Giardia intestinalis* and *Entamoeba histolytica*) are a major public health concern in low- and middle-income countries (LMICs) [1, 2]. More than five billion people are at risk of infection with soil-transmitted helminths and over one billion people are infected, particularly in LMICs [1, 3, 4]. Morbidities due to intestinal parasite infections vary from individual to individual and depend on the type, number and intensity of intestinal parasite and host factors (e.g. preschool- and school-aged children and women of reproductive age are at particular risk) [5, 6]. In 2012, the World Health Organization (WHO) estimated that 270 million preschool-aged children and > 600 million school-aged children lived in areas where helminths and intestinal protozoa are intensively transmitted, and thus warrant interventions [7]. The highest prevalence and intensity of infection with soil-transmitted helminths is usually observed in school-aged children [8]. Chronic helminth infections are manifested in delayed physical and mental development, anaemia and protein-energy malnutrition [3, 5, 9, 10]. Intestinal parasite infections are intimately linked to poverty and inadequate access to water, sanitation and hygiene (WASH). The impact of unsafe WASH on morbidity is particularly severe in malnourished children [11, 12]. The WHO recommends periodic deworming of preschool- and school-aged children as a public health intervention. In places where the prevalence of soil-transmitted helminth exceeds 20%, deworming should be done at least once every year [13].

In Nepal, intestinal parasite infections are widespread and polyparasitism is a concern, as infections with multiple intestinal parasite species can exacerbate morbidity [6, 14]. However, there is a paucity of data on intestinal parasite infection among school-aged children in Nepal. Indeed, only few studies investigated intestinal parasite infections and found considerable variation in the prevalence in school-aged children in different parts of Nepal. The most common helminth species infecting Nepalese children reported in the literature were hookworm, *A. lumbricoides* and *T. trichiura*, while common intestinal protozoa were *E. histolytica* and *G. intestinalis* [15–18]*.* Little is known about infection intensity profiles and underlying risk factors in Nepal.

To fill this gap, a cross-sectional survey was carried out focusing on children aged 8–16 years in two districts of Nepal. Our aim was to determine the prevalence of intestinal parasitic infections and to assess underlying risk factors. The study results were utilized to design complementary school-based interventions to improve the nutritional and health status of schoolchildren. Of note, the study reported here was readily embedded into a multi-country, multi-sectorial project entitled “Vegetables go to School: improving nutrition through agricultural diversification” (Vgts) [19].

## Methods

### Study design, sites and participants

The baseline cross-sectional study was conducted in the Dolakha and Ramcehhap districts, situated in the eastern part of Nepal, covering surface areas of 2191 and 1546 km^2^, respectively. There are 51 village development committees (VDCs) in Dolakha district and 45 in Ramechhap district. Our cross-sectional survey was implemented from March to May 2015, in 32 VDCs in Dolakha district and 8 VDCs in Ramechhap district. The populations in Ramechhap and Dolakha districts were 202,646 and 186,557 people, respectively. Out of 43,910 households in Ramechhap district, 34,902 households had access to piped drinking water, whereas 3429 households depended on uncovered wells and 1242 households used river water for drinking. In Ramechhap district, 35,547 households had access to piped drinking water, while 1495 households depended on uncovered wells and 537 households used river water for drinking. With regard to electricity as a source of lighting, 19,970 and 37,349 households in Ramechhap and Dolakha, respectively, had access. In terms of sanitation facilities, 16,047 and 13,860 households lacked a toilet facility at home in Ramechhap and Dolakha, respectively. With regard to climate, Ramechhap district has a higher percentage of tropical (18.0%) and sub-tropical (42.1%) climate, whereas Dolakha district has a higher percentage of temperate climate (28.5%).

**Fig. 1:**
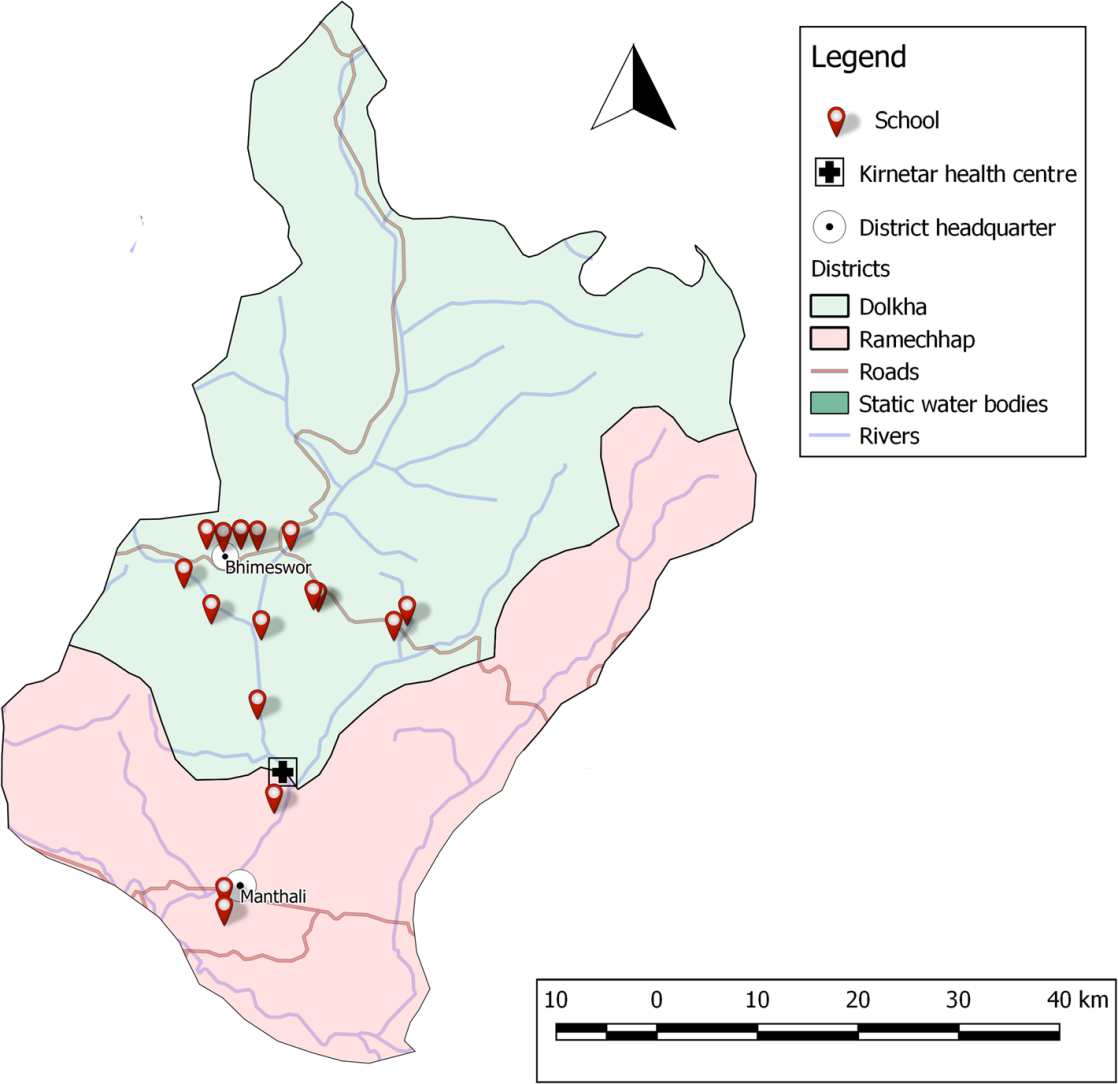
Map of Dolakha and Ramechhap districts in Nepal showing the surveyed schools

The two districts and the surveyed schools were selected as VgtS project sites by national authorities from the National Agricultural Research Council (NARC), Ministry of Education (MoE) and Ministry of Health and Population (MoHP) of Nepal. Sixteen schools were purposely selected within the frame of the VgtS project based on the following criteria: (i) non-boarding public schools teaching at least up to grade 8 with a minimum of 150 students; (ii) schools located in rural or peri-urban areas that can be reached within a maximum of 1 h walking distance from a major road; (iii) no earlier involvement in a school garden programme; (iv) availability of at least 300 m^2^ of land used for gardening and access to a source of water for irrigation; (v) not located in a commercial vegetable growing area; and (vi) the school principal being willing to participate in the project (Fig. 1). Overall, epidemiological data were obtained from 708 children aged 8–16 years. Details of the larger VgtS study and participants’ characteristics have been described elsewhere [20].

### Questionnaire survey

We designed, pre-tested and administered a semi-structured questionnaire to the schoolchildren, their caregivers and school principals. From schoolchildren, demographic data (age and sex) and information regarding knowledge, attitudes and practices (KAP) of personal hygiene were collected. From caregivers, data on socioeconomic status, WASH behaviour and medical history of children in the preceding 2 weeks were collected. From school principals, we obtained data on school-based WASH conditions.

Our questionnaire was developed in English, translated into Nepali and back translated for validation. Pre-testing of the questionnaire was done in selected schools and households outside the study area, characterised by similar geographical and socioeconomic features. Research assistants were trained for data collection. Reliability of the information was ensured by interviewing the schoolchildren and their caregivers in their mother tongue by research assistants who grew up in the study area. For quality control, a principal researcher accompanied each research assistant to three households.

### Stool examination

A pre-labelled plastic container was provided to each schoolchild, along with specific information for collection of at least 10 g of their own morning stool the following day, upon completion of the questionnaire survey [19]. Stool samples were transferred to the laboratory and stored at 4°C pending further analysis [21]. The samples were examined following WHO standard operating procedures (SOPs) [22]. First, approximately 2 g of stool was prepared on a single slide with the saline wet mount method for microscopic detection of intestinal parasites [23]. For quality control, 10% of the slides were re-examined by a senior technician [6]. Egg counts for helminths were compared with the original readings. Whenever discrepancies were observed (e.g. negative *versus* positive results or helminth egg counts differing by over 10%), the slides were re-read and results discussed until agreement was reached [6]. Secondly, duplicate Kato-Katz thick smears using 41.7 mg templates were prepared on microscope slides [6, 24, 25]. Slides were allowed to clear for 30 min prior to examination under a light microscopy at a magnification of 400× by experienced laboratory technicians [6]. Eggs were counted and recorded for each helminth species separately [6, 26]. Infection intensity was expressed as the number of eggs per gram of stool (EPG) by multiplying egg counts with a factor of 24 [27]. Thirdly, a formalin-ether concentration technique was used to detect helminth eggs and larva or cyst of intestinal protozoa [28].

For the analysis of parasitological data, only schoolchildren who provided a sufficient quantity of stool (at least 10 g) and had complete data records were included in the final analysis. Helminth infection intensities were grouped into light, moderate and heavy, according to WHO cut-offs [29]. In brief, light, moderate and heavy classes for *A. lumbricoides* infections were 1–4999 EPG, 5000–49,999 EPG and ≥ 50,000 EPG; for *T. trichiura* classes were 1–999 EPG, 1000–9999 EPG and ≥ 10,000 EPG; and for hookworm, classes were 1–1999 EPG, 2000–3999 EPG and ≥ 4000 EPG. The Kato-Katz technique is characterized by a low diagnostic accuracy for *Enterobius vermicularis*, hence no attempt was made for determining infection intensity of this helminth species [30].

### Clinical examination

Haemoglobin (Hb) was assessed in each child by collecting a fingerpick blood sample using a B-haemoglobin photometer (Hemocue AB; Angelholm, Sweden) [6]. Morbidity information for the 2 weeks prior to the survey was obtained from each child and their caregivers by symptoms recall (e.g. fever, watery diarrhoea, bloody diarrhoea and mucus in stool) and by clinical examination (e.g. hepatomegaly and pale conjunctiva) [6]. An experienced paediatrician conducted clinical examinations. By palpating the liver lobe (left) along the xiphoid-umblicus line (supine position), hepatomegaly was determined [31]. It was classified as present or absent when the left liver lobe was palpable/not palpable [6, 31].

Anaemia was determined according to age-specific Hb levels using WHO cut-offs. Anaemia was defined as a level of Hb < 11.0 g/dl for children aged 8–11 years, < 11.9 g/dl for children aged 12–14 years and < 12.9 g/dl for children aged ≥ 15 years [32]. Severe anaemia was defined as Hb below 8 g/dl, while moderate anaemia was considered as Hb between 8 and 10.9 g/dl [33].

### Water quality analysis

Sampling of water sources and details of the assessment procedure have been described elsewhere [20].

### Statistical analysis

Details of data management and statistical analyses have been described elsewhere [20]. In brief, parasitological data were entered into a MS Excel 2010 spreadsheet (Microsoft; Redmond, WA, USA). Internal consistency checks were performed, and errors removed by comparing the entries with the original laboratory sheets. Schoolchildren with complete data records were included in the final analysis [6]. Children were classified into two age groups (8–12 and 13–16 years) for summary statistics [6].

We employed Chi-square (*χ*^2^) statistics to assess differences in distributions for categorical variables. Risk factors of intestinal parasite infections were assessed using mixed logistic regression models with random intercepts for schools. Children’s age, sex, socioeconomic status of caregivers and district were considered *a priori* as potential confounders, and hence included in multivariate regression models. A new variable for socioeconomic status was created using factor analysis to calculate a wealth index based on household assets, using a k-means procedure. Similarly, a hygiene variable was created using factor analysis with two conceptually similar categorical variables: specific types and frequency of handwashing methods, using a k-means procedure [20]. The socioeconomic and hygiene behaviour of children was categorised as low, medium or good, based on the tertiles of the respective variable [20]. Results were presented as crude and adjusted odds ratios (aORs) with their corresponding 95% confidence intervals (CIs). For the analysis of the outcomes (i) any intestinal parasite infection; (ii) *T. trichiura*; and (iii) *G. intestinalis*, 24 variables were considered as potential predictors based on the extant literature [14]. For all multivariate regression models, *P* < 0.2 in univariate analysis was used as variable entry criterion for the final model. The final model was obtained using backward selection with the same level of *P* < 0.2 [20]. The differences and associations were considered statistically significant if *P* ≤ 0.05 [32]. The population attributable fraction (PAF) was estimated for significantly associated risk factors. Statistical analyses were performed using STATA version 14 (Stata Corporation; College Station, TX, USA) [20].

## Results

### Compliance and characteristics of study population

A total of 708 schoolchildren participated in the study. In the midst of our survey, a major earthquake occurred, damaging most of the houses. As a result, 146 caregivers could not be reached. Hence, only 562 households were retained for our multivariate analysis.

There were 369 female participants (52.1%). The mean age of the schoolchildren was 12.8 years [standard deviation (SD) 1.2 years] with 15.2% aged 8–12 years and the remaining 84.8% aged 13–16 years. There was no statistically significant sex-difference according to age groups (*P* = 0.44). Three-quarters of the schoolchildren belonged to the Tamang (37.9%) and Chhetri ethnicities (37.4%). Most of the Tamang lived in the Dolakha district (77.9%). Regarding caregivers’ characteristics, the mean age was 40.8 years (SD 8.5 years). More than a third (37.4%) of the caregivers had no formal education and 81.5% of the caregivers were engaged in farming as their main occupation. The caregivers’ houses mainly consisted of corrugated iron walls (73.8%), corrugated iron roofs (72.4%) and a mud floor (93.4%). Livestock was kept by 90.2% of the households, of which 45.4% were reported to roam freely inside the house court. Goats were the most commonly present livestock (79.4%), followed by poultry (74.9%). For further details of socio-demographic characteristics, the reader is referred elsewhere [20].

### WASH characteristics of schools and households

All 16 schools had some kind of water infrastructure and 15 of them had access to water at some point during the day. Only 6.3% of school principals reported cleaning the sanitation facility at least once a week. Six out of 16 schools had not implemented a hygiene programme within the past 2 months.

About half (49.0%) of the households had no piped water connected to their house, yet 78.1% of the surveyed households reported having sufficient drinking water throughout the year. Most households (86.5%) did not treat drinking water. Almost a third (29.7%) of the households had no latrine and 25.8% said that they had no soap. Among those household with a latrine (70.1%), most reported to have a water seal latrine (50.4%). Members of 16.8% of the households were either defecating in the bush or river/swamps. Slightly more than half (51.4%) of the households reported that they do not deposit their solid waste safely. Further details of WASH-specific information and behaviour at the unit of the school and household have been reported elsewhere [20].

### Drinking water quality

Contamination of water samples by TTC was observed in 76.9% of the samples collected at schoolchildren’s point-of-use, in 27.4% of the samples obtained from households and in 39.5% of the samples from community water sources (e.g. spring, tap, etc.). We found significant differences in household water samples contaminated with TTC by district (36.4% in Ramechhap *versus* 25.0% in Dolakha; *χ*^2^ = 6.13, *P* = 0.01). For additional details of drinking water quality information at the unit of the school and household, the reader is invited to consult our previous paper [20].

### WASH KAP of schoolchildren and caregivers

Table 1 summarises the KAP results obtained from schoolchildren and their caregivers. On the basis of our questionnaire administered to schoolchildren, 74.7% reported washing their hands with soap and water after defecation, while 72.6% reported doing so before eating and 58.0% after playing. Approximately 4% of the children reported not using the latrine at school. One out of 100 children reported defecating either in the fields around their home or behind the latrines at home or school. The overall hygiene behaviour of the children, including the occasions and materials used for hand washing, differed significantly by district (*χ*^2^ = 19.42, *P* < 0.001), while no significant differences were found with regard to caregivers sanitary practices (*χ*^2^ = 2.70, *P* = 0.26). The majority of surveyed schoolchildren (90.0%) reported that they drank water from the sources provided at the school. Only 10.2% of children reported that intestinal parasite infections were transmitted by dirty water. Around 8% of the children had a misconception that intestinal parasite infection may occur after eating chocolates or other sugary products.

**Table 1:** Questionnaire findings on KAP of schoolchildren and water quality results in schools in Dolakha and Ramechhap districts of Nepal between March and May 2015

Children	Overall (*N* = 708)	Dolakha (*n* = 555)	Ramechhap (*n* = 153)	*P*-value^a^
KAP indicators	*N* (%)	*n* (%)	*n* (%)	
Knowledge on handwashing				
Before eating	525 (74.2)	427 (76.9)	98 (64.1)	**0.01**
After eating	434 (61.3)	357 (64.3)	77 (50.3)	**0.01**
After playing	422 (59.6)	345 (62.2)	77 (50.3)	**0.01**
After defaecation	534 (75.4)	427 (76.9)	107 (69.9)	0.08
Handwashing practices				
Before eating	0 (0)	0 (0)	0 (0)	–
After eating	473 (66.8)	387(69.7)	86 (56.2)	**0.01**
After playing	428 (60.6)	345 (62.2)	83 (54.3)	0.08
After defaecation	641 (90.5)	505 (91.0)	136 (88.9)	0.43
Handwashing with				
Water	687 (97.0)	540 (97.3)	147 (96.1)	0.43
Ash	17 (2.4)	12 (2.2)	5 (3.3)	0.43
Mud, soil	4 (0.6)	4 (0.7)	0 (0)	0.29
Soap	689 (97.3)	539 (97.1)	150 (98.0)	0.53
Why wash hands with soap?				
Remove germs	196 (27.7)	137 (24.7)	59 (38.6)	–
Prevent illness	365 (51.5)	306 (55.1)	59 (38.6)	
Clean hands	94 (13.3)	61 (11.0)	33 (21.6)	
Don’t know	78 (11.0)	65 (11.7)	13 (8.5)	
Hygiene				
Better category	261 (36.9)	225 (40.5)	36 (23.5)	**0.01**
Middle category	211 (29.8)	165 (29.7)	46 (30.1)	
Poor category	236 (33.3)	165 (29.7)	71 (46.4)	
Sanitary practices at school				
Use latrine at school	679 (95.9)	543 (97.8)	136 (88.9)	**<0.001**
No latrine use	29 (4.1)	12 (2.2)	17 (11.1)	
Reasons for not using latrine (*n* = 17)				
Dirty	12 (70.6)	1 (50.0)	11 (73.3)	0.09
No soap	1 (5.9)	0 (0)	1 (6.7)	
Not functional	2 (11.8)	0 (0)	2 (13.3)	
Other	1 (5.9)	0 (0)	1 (6.7)	
No response	1 (5.9)	1 (50.0)	0 (0)	
Defaecation location if not using latrine (*n* = 17)				
Bush	13 (76.5)	2 (100)	11 (73.3)	0.40
Behind the latrine	4 (23.5)	0 (0)	4 (26.7)	
Opinion on whether dirty water causes illness				
Yes	694 (98.0)	544 (98.0)	150 (98.0)	0.99
No	14 (2.0)	11 (2.0)	3 (2.0)	
Knowledge on illnesses caused by dirty water				
Diarrhoea	458 (64.7)	377 (67.9)	81 (52.9)	**<0.001**
Cholera	138 (19.5)	108 (19.5)	30 (19.6)	0.97
Skin irritation	47 (6.6)	31 (5.6)	16 (10.5)	**0.03**
Icterus	12 (1.7)	11 (2.0)	1 (0.7)	0.26
Typhus	41 (5.8)	38 (6.9)	3 (2.0)	**0.02**
Malaria	14 (2.0)	12 (2.2)	2 (1.3)	0.50
Eye irritation/disease	9 (1.3)	9 (1.6)	0 (0)	0.11
Worms, parasites	72 (10.2)	58 (10.5)	14 (9.2)	0.64
Perception on becoming sick by not washing hands by schoolchildren				
Yes	696 (98.3)	543 (97.8)	153 (100)	0.50
No	5 (0.7)	5 (0.9)	0 (0)	
Not sure	7 (1.0)	7 (1.3)	0 (0)	
Drinking water				
Drinking water from school	637 (90.0)	535 (96.4)	102 (66.7)	**<0.001**
Bringing water from home	102 (14.4)	67 (12.1)	35 (22.9)	**0.01**

^a^*P*-values calculated by *χ*^2^ test; values in boldface indicate statistically significant differences

More than half of the caregivers reported using a private tap as their main drinking water source regardless of the season. Most households reported fetching their drinking water in a metal (47.0%) or a plastic container (45.9%) and 61.8% stated that they wash these drinking water containers daily with soap. Only 19.8% reported treating drinking water before consumption. A significant difference was observed in drinking water treatment (22.0% in Ramechhap *versus* 11.3% in Dolakha; *χ*^2^ = 9.25, *P* = 0.01). Most of the caregivers (70.6%) had heard about intestinal parasites. Preventive measures, as reported by caregivers, included cutting fingernails (37.7%), drinking clean water (32.7%), washing fruits and vegetables (16.7%) and wearing shoes (9.3%).

### Results from the clinical survey

Table 2 presents the frequency of reported symptoms of schoolchildren, stratified by sex, age group and district. Of the children surveyed, 11.5% reported being sick the day before the survey; the most frequently reported symptom was fever (31.0%). Watery diarrhoea, mucus in the stool and bloody diarrhoea were reported by 22.4, 2.8 and 1.4% of the caregivers, respectively. There was a significant difference in self-reported fever by district (32.9% in Dolakha *versus* 23.7% in Ramechhap; *χ*^2^ = 3.07, *P* = 0.05). The prevalence of reported diarrhoea was significantly higher in children aged 8–12 years compared to their older counterparts (*P* = 0.01). The most frequently diagnosed clinical sign was pale conjunctiva (9.8%).

**Table 2:** Frequency of clinical outcomes obtained from physical examination of children in Dolakha and Ramechhap districts, Nepal, between March and May 2015, stratified by sex, age group and district

Clinical outcomes	Overall (*N* = 562)	Sex				Age group (years)				District			
		Male (*n* = 280)	Female (*n* = 282)	*χ* ^2^	*P*	8–12 (*n* = 122)	13–16 (*n* = 440)	*χ* ^2^	*P*	Dolakha (*n* = 444)	Ramechhap (*n* = 118)	*χ* ^2^	*P*
	*N* (%)	*n* (%)	*n* (%)			*n* (%)	*n* (%)			*n* (%)	*n* (%)		
Symptoms in past two weeks													
Fever	174 (31.0)	84 (30.0)	90 (31.9)	0.24	0.62	37 (30.3)	137 (31.1)	0.02	0.86	146 (32.9)	28 (23.7)	3.07	**0.05**
Diarrhoea	126 (22.4)	66 (23.6)	60 (21.3)	0.43	0.51	38 (31.1)	88 (20.0)	6.82	**0.01**	104 (23.4)	22 (18.6)	1.02	0.27
Blood in stool	8 (1.4)	5 (1.8)	3 (1.1)	0.52	0.47	2 (1.6)	6 (1.4)	0.05	0.82	4 (0.9)	4 (3.4)	4.01	**0.04**
Mucus in stool	16 (2.8)	5 (1.8)	11 (3.9)	2.27	0.13	5 (4.1)	11 (2.5)	0.88	0.35	15 (3.4)	1 (0.8)	2.02	0.14
Physical examination													
Hepatomegaly	1 (0.2)	1 (0.4)	0 (0)	1.00	0.32	1 (0.8)	0 (0)	3.61	**0.05**	1 (0.2)	0 (0)	0.03	0.60
Pale conjunctiva	55 (9.8)	24 (8.6)	31 (11.0)	0.93	0.33	16 (13.1)	39 (8.9)	1.96	0.16	43 (9.7)	12 (10.2)	0.02	0.88

*P*-values were obtained by *χ*^2^ test; values in boldface indicate statistically significant differences

Overall, 23.6% of the children were found to be anaemic with no significant difference by sex (56.1% in females *versus* 48.4% in males; *χ*^2^ = 2.39, *P* = 0.12) nor age group (26.2% in children aged 8–12 years *versus* 22.7% in children aged 13–16 years; *χ*^2^ = 0.65, *P =* 0.42). Significant differences were observed for anaemia by district (33.1% in Ramechhap *versus* 21.0% in Dolakha; *χ*^2^ = 7.60, *P* = 0.006). The mean Hb concentration found was 12.6 g/dl (SD 1.2 g/dl), ranging from a minimum of 7.7 g/dl to a maximum of 16.5 g/dl.

### Prevalence and intensity of intestinal parasite infections

**Fig. 2:**
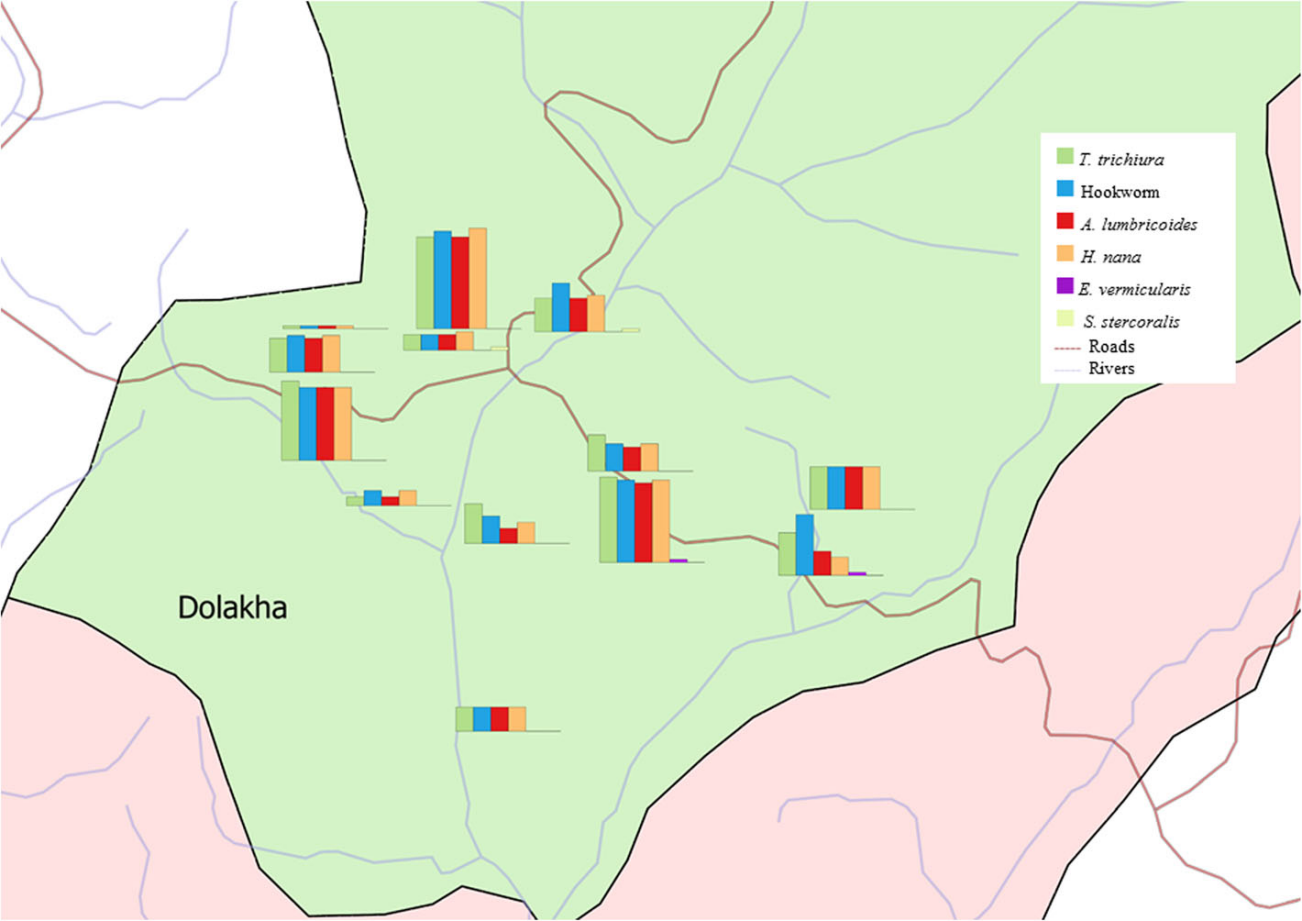
Intestinal parasites in 13 schools of Dolakha district, Nepal in March-May 2015

**Fig. 3:**
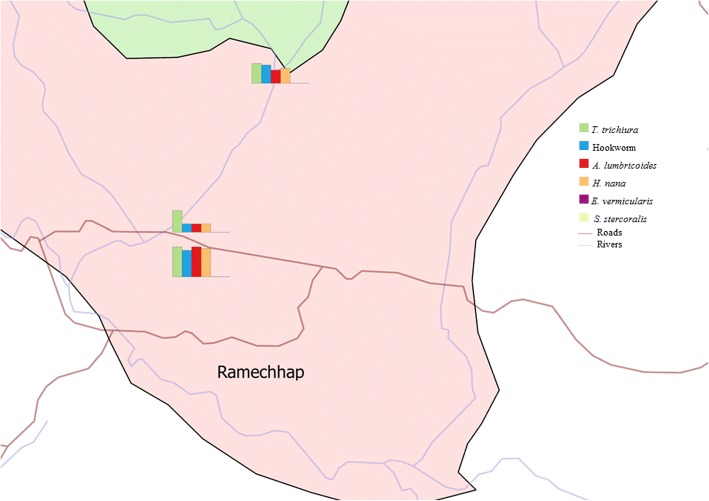
Intestinal parasites in three schools of Ramechhap district, Nepal in March-May 2015

Table 3 summarises the overall prevalence of intestinal parasite infection, stratified by sex, age group and district. The overall prevalence of intestinal parasite infection considering both soil-transmitted helminths and intestinal protozoa was 39.7%. The predominant helminth species were *T. trichiura* (30.9%) and hookworm (30.2%), followed by *A. lumbricoides* (26.1%). *Enterobius vermicularis* and *Strongyloides stercoralis* were detected only rarely (0.4 and 0.3%, respectively) (Figs. 2, 3). Most of the soil-transmitted helminth infections were of light intensity. The cumulative prevalence of intestinal protozoa infection was 30.5% with *G. intestinalis* (30.5%) identified as the predominant species. Occurrence of double or triple infections was frequent. For example, 13.8% of the study participants had a triple infection with hookworm, *T. trichiura* and *G. intestinalis*.

**Table 3:** Intestinal parasitic infections of schoolchildren in Dolakha and Ramechhap districts of Nepal between March and May 2015

Parasite	Prevalence (*N* = 708)	Sex		*χ* ^2^	*P*	Age group		*χ* ^2^	*P*	District		*χ* ^2^	*P*
		Male (*n* = 339)	Female (*n* = 369)			8–12 years (*n* = 108)	13–16 years (*n* = 600)			Dolakha (*n* = 555)	Ramechhap (*n* = 153)		
	*N* (%)	*n* (%)	*n* (%)			*n* (%)	*n* (%)			*n* (%)	*n* (%)		
Nematodes													
*Ascaris lumbricoides*	185 (26.1)	87 (25.7)	98 (26.6)	0.07	0.79	21 (19.4)	164 (27.3)	2.95	0.09	154 (27.8)	31 (20.3)	3.48	0.06
*Trichuris trichiura*	219 (30.9)	109 (32.2)	110 (29.8)	0.45	0.50	27 (25.0)	192 (32.0)	2.10	0.15	176 (31.7)	43 (28.1)	0.73	0.39
Hookworm	214 (30.2)	99 (29.2)	115 (31.2)	0.32	0.57	27 (25.0)	187 (31.2)	1.65	0.12	182 (32.8)	32 (20.9)	8.02	**0.01**
*Strongyloides stercoralis*	2 (0.3)	1 (0.3)	1 (0.3)	0.01	0.95	0 (0)	2 (0.3)	0.36	0.55	2 (0.4)	0 (0)	0.55	0.46
*Enterobius vermicularis*	2 (0.4)	1 (0.4)	1 (0.4)	0.01	0.96	0 (0)	2 (0.5)	0.39	0.53	2 (0.5)	0 (0)	0.62	0.43
Cestodes													
*Hymenolepis nana*	195 (27.5)	90 (26.6)	105 (28.5)	0.32	0.57	24 (22.2)	171 (28.5)	1.81	0.18	164 (29.6)	31 (20.3)	5.18	**0.02**
Total faecal-oral transmitted helminths	250 (35.3)	120 (35.4)	130 (35.2)	0.01	0.96	31 (28.7)	219 (36.5)	2.44	0.12	203 (36.6)	47 (30.7)	1.80	0.18
Intestinal protozoa													
*Giardia intestinalis*	216 (30.5)	101 (29.8)	115 (31.2)	0.16	0.69	22 (20.4)	194 (32.3)	6.18	**0.01**	176 (31.7)	40 (26.1)	1.75	0.19
Total intestinal protozoa	216 (30.5)	101 (29.8)	115 (31.2)	0.16	0.69	22 (20.4)	194 (32.3)	6.18	**0.01**	176 (31.7)	40 (26.1)	1.75	0.19

*P*-values were obtained by χ2 test; values in boldface indicate statistically significant differences

The difference between the overall prevalence of intestinal parasite infection by district was not significant (43.5% in Dolakha *versus* 36.4% in Ramechhap; *χ*^2^ = 1.89, *P* = 0.17). The overall prevalence of intestinal parasite infection was similar in males compared to females (40.4% *versus* 39.0%; *χ*^2^ = 0.14, *P* = 0.71).

### Risk factors for intestinal parasite infections

Results from the logistic regression analyses are given in Table 4, Additional file 1: Table S1 and Additional file 2: Table S2. Age was significantly associated with the overall intestinal parasite infection. Children aged 8–12 years had lower odds of infection compared with their older counterparts (OR 0.61; 95% CI: 0.38–0.99, *P =* 0.04) (Table 4). Schoolchildren from households who did not have soap for handwashing were at a higher odds of intestinal parasite infection (aOR 1.81; 95% CI: 1.13–2.89, *P* = 0.01)*.* No statistically significant association was found between intestinal parasite infection and sources of drinking water, containers used for fetching water or treatment of water. Living in a household without sanitation facilities was negatively associated with *T. trichiura* (aOR 0.52; 95% CI: 0.29–0.92, *P* = 0.02; Additional file 1: Table S1). Holding domestic animals outside the household was negatively associated with *G. intestinalis* and the association was statistically significant (aOR 0.52; 95% CI: 0.33–0.83; *P* = 0.01; Additional file 2: Table S2).

**Table 4:** Results from univariate and multivariate logistic regression analyses for parasitic infection in two districts of Nepal in March-May 2015. The multivariate global model includes a random intercept at the level of school adjusting sex, age and district, where all the variables were assessed one by one and retained for the global model if their *P*-value is < 0.2 in univariate analysis (values in bold). The final model was obtained by using backward selection with the same level of 0.2

Risk factor	*N* = 562	Any parasitic infection (*n* = 236)					
		Univariate analysis			Multivariate analysis		
	*n* (%)	OR	95% CI	*P*	aOR	95% CI	*P* ^a^
Sex							
Female	282 (50.2)	1.00			1.00		
Male	280 (49.8)	1.10	0.77–1.58	0.60	1.09	0.75–1.59	0.64
Age							
13–16 years	440 (78.3)	1.00					
8–12 years	122 (21.7)	0.65	0.42–1.03	**0.07**	0.61	0.38–0.99	**0.04**
District							
Dolakha	444 (79.0)	1.00			–		
Ramechhap	118 (21.0)	0.77	0.25–2.39	0.65	0.93	0.30– 2.90	0.90
Hygiene behaviour							
Lower category	245 (31.1)	1.00					
Middle category	142 (25.3)	0.87	0.55–1.38	0.56			
Higher category	175 (31.1)	0.89	0.57–1.37	0.59			
Drinking water consumption							
From school	491 (87.4)	1.00					
From home	71 (12.6)	0.85	0.48–1.49	0.56			
Water risk behaviour							
Playing (yes *vs* no)	173 (30.8)	1.12	0.76–1.66	0.57			
Fishing (yes *vs* no)	68 (12.1)	1.04	0.59–1.82	0.89			
Laundry (yes *vs* no)	199 (35.4)	1.23	0.82–1.85	0.32			
Domestic chores (yes *vs* no)	142 (25.3)	1.18	0.75–1.87	0.48			
Sanitary practices							
Using latrine at school (yes *vs* no)	550 (97.9)	0.69	0.26–1.85	0.47			
Ethnicity of children							
Tamang	13 (37.9)	1.00					
Brahmin	101 (18.0)	1.08	0.62–1.90	0.78	1.20	0.67–2.17	0.53
Chhetri	210 (37.4)	0.97	0.61–1.53	0.89	1.00	0.62–1.62	0.99
Newar	33 (5.9)	1.32	0.55–3.16	0.54	1.29	0.52–3.17	0.58
Janajati	5 (0.9)	3.60	0.50–25.97	0.20	3.98	0.52–30.54	0.18
Caregiver’s education							
Never went to school	210 (37.4)	1.00					
Primary education	144 (25.6)	0.91	0.55–1.51	0.71	1.00	0.60–1.69	1.00
Secondary education	143 (25.4)	0.98	0.54–1.79	0.96	1.27	0.67–2.41	0.47
Higher education	65 (11.6)	0.70	0.31–1.62	0.41	0.90	0.38–2.16	0.82
Caregiver’s occupation							
Farmer	458 (81.5)	1.00			–		
Public services	39 (6.9)	0.62	0.29–1.37	0.24	0.55	0.24–1.25	0.15
Business	40 (7.1)	0.87	0.39–1.93	0.73	0.87	0.37–2.04	0.75
Other	25 (4.5)	0.36	0.13–0.96	**0.04**	0.35	0.13–0.99	0.05
Socioeconomic status							
Poor	298 (53.0)	1.00					
Average	215 (38.3)	1.34	0.91–1.98	**0.14**	1.29	0.86–1.92	0.22
High	49 (8.7)	1.02	0.53–1.99	0.95	0.88	0.45–1.76	0.73
Drinking water in dry season							
Private tap	287 (51.1)	1.00					
Protected spring	13 (2.3)	1.84	0.50–6.81	0.36			
Public tap	36 (6.4)	1.31	0.51–3.26	0.53			
Other	226 (40.2)	1.16	0.75–1.79	0.50			
Drinking water in rainy season							
Private tap	285 (50.7)	1.00					
Protected spring	1 (0.2)	na					
Public tap	44 (7.8)	1.12	0.49–2.54	0.79			
Other	232 (41.3)	1.31	0.86–2.01	0.21			
Water sufficiency for drinking and household chores	439 (78.1)	0.85	0.50–1.42	0.53			
Frequency of washing drinking water container with soap							
Daily	347 (61.7)	1.00					
Never	40 (7.1)	1.41	0.65–3.06	0.39			
Weekly	175 (31.1)	1.36	0.85–2.17	0.20			
Container for fetching water							
Metal	264 (31.1)	1.00					
Plastic	258 (61.7)	1.22	0.79–1.89	0.37			
Clay pot	40 (7.1)	0.56	0.23–1.37	0.21			
Status of drinking water container							
Covered	417 (74.2)	1.00					
Uncovered	145 (25.8)	1.01	0.64–1.61	0.96			
Drinking water container used for other activity (yes *vs* no)	111 (19.8)	1.35	0.78–2.33	0.29			
Water treatment prior to consumption (yes *vs* no)	76 (13.5)	0.74	0.41–1.35	0.33			
Water contamination with thermotolerant coliform (yes *vs* no)	154 (27.4)	1.06	0.70–1.60	0.79			
Sanitation in the household							
Water seal latrine	283 (50.4)	1.00					
No latrine	168 (29.9)	0.92	0.55–1.54	0.76			
Open pit latrine with slab	97 (17.3)	0.75	0.41–1.35	0.33			
Open pit latrine without slab	14 (2.5)	1.07	0.33–3.46	0.90			
Soap for handwashing available (no *vs* yes)	417 (74.2)	1.85	1.18–2.92	**0.01**	1.81	1.13–2.89	**0.01**
Waste disposal (yes *vs* no)	273 (48.6)	1.03	0.70–1.52	0.88			
Domestic animals							
Possession of domestic animals (yes *vs* no)	507 (90.2)	1.07	0.57–2.02	0.83			
Animals held outside the house (yes *vs* no)	307 (54.6)	0.89	0.57–1.39	0.62			

^a^*P*-values < 0.05 in multivariate analyses are marked in boldface

Population-attributable risk analysis suggested that an estimated 11.3% of intestinal parasite infections might have been averted through handwashing with soap. An estimated 15.6% of *G. intestinalis* infections might have been averted if animals were not allowed to roam freely inside the household.

## Discussion

Our data confirm that intestinal parasite infections are prevalent among schoolchildren in Dolakha and Ramechhap districts of eastern Nepal, which contrasts with a decline of intestinal parasite infections reported in other districts of Nepal [34]. Indeed, stool samples subjected to duplicate Kato-Katz thick smears and single wet mount and formalin-ether concentrated methods showed that one out of three schoolchildren were infected with at least one helminth and/or pathogenic intestinal protozoa infection. Soil-transmitted helminth infections were slightly more prevalent than intestinal protozoa infections. The predominant helminth species were *T. trichiura* and hookworm, while *G. intestinalis* was the predominant intestinal protozoa infection. Our observations contrast with previous epidemiological surveys that revealed intestinal protozoa being more prevalent than helminths [18, 35]. Unsafe WASH exacerbates parasite infections in general and helminth infections in particular [12, 36]. This might have governed the high prevalence of intestinal parasite infections in a setting where almost one-third of households reported not having any sanitation facilities at home and almost one-third of the children reported not washing their hands with soap after defecation. Our study also showed a considerable number of schoolchildren infected with *A. lumbricoides*, which is in line with studies carried out in another part of Nepal [37]. This could be due to the fact that transmission of *A. lumbricoides* is through the faecal-oral route with re-infections occurring quickly after treatment [38]. Since open defecation is widely practised, efforts must be made to improve sanitation practices, which are likely to have ramifications on soil-transmitted helminthiasis, intestinal protozoa infections and other neglected tropical diseases [12, 36, 39].

Our study revealed that multiparasitism was common, as reported elsewhere in Asia [14]. Indeed, we found that 39.7% of the children harboured multiple species of intestinal parasites concurrently, which is a major public health concern as multiple species parasite infections may increase susceptibility to other infections [40]. Reasons that might explain the high level of multiparasitism are the low socioeconomic status and lack of awareness of the surveyed schoolchildren and their caregivers about the transmission of intestinal parasites and how such infections can be prevented. Interestingly, most of the children diagnosed with a single helminth species were characterised by low infection intensity profiles, which is in contrast to findings from the baseline cross-sectional survey of the VgtS study in Burkina Faso. Indeed, in Burkina Faso, many of the infected children showed moderate helminth intensities and there was a higher percentage of children with intestinal protozoa infections compared to our study in Nepal [32]. Yet, it should be noted that low-intensity helminth infections might negatively impact on children’s health and wellbeing [41–43]. Despite this, we are not aware of any large-scale, regular deworming activities carried out in our study area, although our findings suggest that such interventions are indicated.

Our study also determined risk factors for intestinal parasite infections, including the influence of age, sex and study setting. Of particular note is the negative association between any intestinal parasite infection and age with an adjusted OR below one. With regard to sex, both males and females showed similar infection rates, which corresponds with a study conducted in the Lalitpur district [44]. Behavioural and socioeconomic factors might explain the observed similarity. We found a significantly higher prevalence of the overall intestinal parasite infection and *G. intestinalis* among children whose caregivers were involved in farming activities. These observations are in line with a previous study conducted in the district of Lalitpur, where children belonging to farmer parents were most commonly infected [44]. Interestingly, our study revealed a significant negative association between *T. trichiura* infection and having no latrine, compared to having a water seal latrine in the household. There are no immediate explanations for this negative relationship, but other confounding factors such as hygiene behaviour may account for this observation. Even without improved sanitary facilities, adequate hygiene practices could make a difference in children’s infection status with intestinal parasites. Further in-depth studies in the two study districts are warranted to deepen the understanding of health benefits of improved WASH. For instance, we found a significant association between those children living in households that did not have soap for handwashing after defecation and intestinal parasite infections, which corroborates prior studies conducted in Nepal [16]. Furthermore, in our study, higher odds were found for intestinal parasite infection for children from households without soap for handwashing, as compared to those households that had soap for handwashing. Additionally, we found a significant association between domestic animals kept outside the households, as there were lower odds of infection with *G. intestinalis* among those children. A similar association between children’s proximity to livestock and *G. intestinalis* infection was found in a study conducted in rural India [45].

We did not find any significant associations between intestinal parasite infections and clinical signs, but this observation requires further in-depth studies on whether and to what extent intestinal parasite infections can impact on children’s health and wellbeing [6]. Without a deeper mechanistic understanding of how intestinal parasite infections might influence children’s health and development, the effectiveness of parasitic disease control programmes are compromised [46].

We observed a high level of water contamination with TTC, which is an indicator of pollution of drinking water sources or drinking water vessels by organic means or domestic effluents. This might illustrate the inadequacy of the cleanliness of the storage containers and drinking vessels. Additionally, this may be due to constructional defects of water infrastructures, poor sanitation and the existence of animal or human waste in close proximity to open freshwater sources. Prior to the April 2015 earthquake, drinking water was mainly supplied by private pipes in Dolakha district and by gravity water supply schemes in Ramechhap district. After the earthquake, the proportion of supply by private pipe has dropped in Dolakha district from 56 to 47%, while only 44% of the gravity water supply remained functional after the earthquake in Ramechhap district [47, 48]. The communities in our study areas had access to water from springs, rivers and private pipes with shared taps. However, we did not find a significant association between TTC in drinking water and intestinal parasite infections, which is in line with observation by the VgtS project in Burkina Faso [32]*.*

Our study has several limitations that are offered for consideration. First, our data were obtained from a relatively small number of schools in two districts of Nepal, and hence wider generalisation is not possible. Secondly, the number of schools selected in Dolakha was considerably higher than in Ramechhap district (13 *versus* 3), which is a problem for elucidating regional differences. Thirdly, children’s age was determined by verbal reporting of children and their caregivers without definitive proof (such as a birth certificate). Fourthly, the diagnosis of intestinal parasites was based on single stool samples that were subjected to a suite of methods. Clearly, examination of multiple consecutive stool samples and triplicate or quadruplicate (rather than duplicate) Kato-Katz thick smears would have resulted in higher diagnostic sensitivity [6, 26, 49]. Employing techLab enzyme-linked immunosorbent assays (ELISAs), and polymerase chain reaction (PCR) might have revealed additional infections not detected by our methods. However, such tests were not available. Fifthly, we used an Oxfam Delagua kit for water quality assessment. An important limitation of this kit is that it does not detect the presence of parasitic elements. Sixthly, anaemia can be caused by multiple and complex factors, and hence it must be noted that by using a B-haemoglobin photometer device for Hb measurement, the identification of the exact type of anaemia was not possible [6, 50, 51]. Seventhly, due to a major earthquake that occurred in the midst of our cross-sectional survey in April 2015, we failed to obtain the targeted number of 800 children. Indeed, we were unable to collect data in three schools and the final number of children in the 16 surveyed schools was 708. This decreased the statistical power and precision of our data.

Despite these shortcomings, a major strength of our study is the appraisal of morbidity including self-reported signs and symptoms (e.g. fever, watery diarrhoea, bloody diarrhoea and mucus in stool), clinical morbidities (e.g. hepatomegaly and pale conjunctiva), and assessment of anaemia, as indirectly determined by quantification of Hb levels. An additional strength is the analytical approach taken (i.e. multivariate analysis) that allowed adjustments of potential confounders such as demographic, socioeconomic, regional differences and personal behavioural information. Moreover, although the diagnostic approach consisted of the collection of only a single stool sample per child, it was complemented by multiple diagnostic methods (i.e. wet mount, formal-ether concentration and Kato-Katz methods), which enhanced diagnostic sensitivity [6].

## Conclusions

We conclude that intestinal parasite infections are a public health problem in Nepal. We found a high prevalence of soil-transmitted helminths and intestinal protozoa infections among children aged 8–16 years. Our observations and results call for specific preventive measures and control interventions targeting schoolchildren. We believe that the morbidities caused by intestinal parasite infections can be overcome or prevented if adequate integrated control measures were to be promoted and implemented, such as the provision of soap for handwashing and regular deworming. These measures could minimise the burden of intestinal parasite infections in the two study districts. Additionally, emphasis should be placed on health promotion programmes at a regional level. The findings from our study provide setting-specific information for designing and implementing preventive programmes for overcoming the burden of intestinal parasite infections in Nepal and other similar countries in Southeast Asia.

## Additional files

Additional file 1: **Table S1.** Results from univariate and multivariate logistic regression analyses for *Trichuris trichiura*. The multivariate global model includes a random intercept at the level of school adjusting sex, age, district where all the variables were assessed one by one and retained for the global model if their *P*-value is < 0.2. The final model was obtained by using backward selection with the same level of 0.2. (DOCX 35 kb)

Additional file 2: **Table S2.** Results from univariate and multivariate logistic regression analyses for *Giardia intestinalis*. The multivariate global model includes a random intercept at the level of school adjusting sex, age, district where all the variables were assessed one by one and retained for the global model if their *P*-value is < 0.2. The final model was obtained by using backward selection with the same level of 0.2. (DOCX 36 kb)

## Abbreviations

aOR: Adjusted odds ratio
CI: Confidence interval
DHS: Demographic and Health Service
EKNZ: Ethikkommission Nordwest- and Zentralschweiz
EPG: Eggs per gram of stool
Hb: Haemoglobin
KAP: Knowledge, attitudes and practices
LMICs: Low- and middle-income countries
MoE: Ministry of Education
MoHP: Ministry of Health and Population
ODK: Open data kit
OR: Odds ratio
SD: Standard deviation
SDG: Sustainable Development Goal
SOP: Standard operating procedure
Swiss TPH: Swiss Tropical and Public Health Institute
TTC: Thermotolerant coliforms
VDC: Village development committee
VgtS: Vegetables go to School
WASH: Water, sanitation and hygiene
WHO: World Health Organization
