# Supplementary material for: Intestinal parasite infections and associated risk factors among schoolchildren in Dolakha and Ramechhap districts, Nepal: a cross-sectional study
Source: Parasit Vectors. 2018 Sep 29;11:532. doi: 10.1186/s13071-018-3105-0 (PMC6162948; doi:10.1186/s13071-018-3105-0)
Supplement: Supplementary file 1 — Table S1. Results from univariate and multivariate logistic regression analyses for Trichuris trichiura. The multivariate global model includes a random intercept at the level of school adjusting sex, age, district where all the variables were assessed one by one and retained for the global model if their P-value is < 0.2. The final model was obtained by using backward selection with the same level of 0.2. (DOCX 35 kb) [file 13071_2018_3105_MOESM1_ESM.docx]

Additional file 1: **Table S1** Results from univariate and multivariate logistic regression analyses for *Trichuris trichuira*. The multivariate global model includes a random intercept at the level of school adjusting sex, age, district where all the variables were assessed one by one and retained for the global model if their *P*-value is < 0.2. The final model was obtained by using backward selection with the same level of 0.2.

| **Risk factor** | | ***Trichuris trichuira* (n=185)** | | | | | | | | | | | | | | | | |
| --- | --- | --- | --- | --- | --- | --- | --- | --- | --- | --- | --- | --- | --- | --- | --- | --- | --- | --- |
|  |  | **Univariate analysis** | | | | | | | | | | | **Multivariate analysis** | | | | | |
|  |  | **OR** | | | | **95% CI** | | | ***P*** | | | | **aOR** | | **95% CI** | | ***P*** | |
| Sex | |  | | | |  | | |  | | | |  | |  | |  | |
| Male | | 1.15 | | | | 0.79-1.67 | | | 0.47 | | | | 1.10 | | 0.75-1.62 | | 0.63 | |
| Female | | 1.00 | | | |  | | |  | | | |  | |  | |  | |
| Age | |  | | | |  | | |  | | | |  | |  | |  | |
| 8-11 years | | 0.76 | | | |  | | | 0.26 | | | | 0.74 | | 0.45-1.21 | | 0.23 | |
| >12 years | |  | | | |  | | |  | | | |  | |  | |  | |
| District | |  | | | |  | | |  | | | |  | |  | |  | |
| Dolakha | | 1.00 | | | |  | | |  | | | |  | |  | |  | |
| Ramechhap | | 0.90 | | | | 0.29-2.80 | | | 0.86 | | | | 0.87 | | 0.28-2.64 | | 0.80 | |
| Hygiene behavior | |  | | | |  | | |  | | | |  | |  | |  | |
| Lower category | | 1.00 | | | |  | | |  | | | |  | |  | |  | |
| Middle category | | 0.96 | | | | 0.61-1.52 | | | 0.87 | | | |  | |  | |  | |
| Higher category | | 1.07 | | | | 0.66-1.71 | | | 0.79 | | | |  | |  | |  | |
| Drinking water consumption | |  | | | |  | | |  | | | |  | |  | |  | |
| From school | | 1.00 | | | |  | | |  | | | |  | |  | |  | |
| From home | | 0.80 | | | | 0.44-1.44 | | | 0.45 | | | |  | |  | |  | |
| Water risk behavior | |  | | | |  | | |  | | | |  | |  | |  | |
| Playing (yes vs no) | | 1.12 | | | | 0.74-1.68 | | | 0.59 | | | |  | |  | |  | |
| Fishing (yes vs no) | | 1.11 | | | | 0.62-1.97 | | | 0.73 | | | |  | |  | |  | |
| Laundry (yes vs no) | | 1.06 | | | | 0.69-1.61 | | | 0.80 | | | |  | |  | |  | |
| Domestic chores (yes *vs* no) | | 1.02 | | | | 0.63-1.64 | | | 0.94 | | | |  | |  | |  | |
| Sanitary practices | |  | | | |  | | |  | | | |  | |  | |  | |
| Using latrine at school (yes *vs* no) | | 0.95 | | | | 0.34-2.64 | | | 0.92 | | | |  | |  | |  | |
| Ethnicity of children | |  | | | |  | | |  | | | |  | |  | |  | |
| Brahmin | | 1.07 | | | | 0.60-1.88 | | | 0.83 | | | | 1.09 | | 0.60-1.97 | | 0.78 | |
| Chhetri | | 1.09 | | | | 0.68-1.74 | | | 0.73 | | | | 1.09 | | 0.67-1.79 | | 0.72 | |
| Newar | | 1.36 | | | | 0.54-3.40 | | | 0.52 | | | | 1.30 | | 0.51-3.32 | | 0.58 | |
| Tamang | | 1.00 | | | |  | | |  | | | |  | |  | |  | |
| Janajati | | 1.92 | | | | 0.26-13.97 | | | 0.52 | | | | 2.17 | | 0.28-16.8 | | 0.46 | |
| Caregiver`s education | |  | | | |  | | |  | | | |  | |  | |  | |
| Never went school | | 1.00 | | | |  | | |  | | | |  | |  | |  | |
| Primary education | | 0.79 | | | | 0.46-1.35 | | | 0.39 | | | |  | |  | |  | |
| Secondary education | | 1.18 | | | | 0.64-2.18 | | | 0.60 | | | |  | |  | |  | |
| Higher education | | 0.77 | | | | 0.33-1.82 | | | 0.56 | | | |  | |  | |  | |
| Caregiver`s occupation | |  | | | |  | | |  | | | |  | |  | |  | |
| Farmer | | 1.00 | | | |  | | |  | | | |  | |  | |  | |
| Public services | | 0.56 | | | | 0.23-1.37 | | | 0.21 | | | | 0.43 | | 0.17-1.11 | | 0.08 | |
| Business | | 1.15 | | | | 0.51-2.59 | | | 0.74 | | | | 1.10 | | 0.46-2.64 | | 0.83 | |
| Other | | 0.44 | | | | 0.16-1.24 | | | **0.12** | | | | 0.37 | | 0.13-1.08 | | 0.07 | |
| Socioeconomic status | |  | | | |  | | |  | | | |  | |  | |  | |
| High | | 0.98 | | | | 0.49-1.96 | | | 0.96 | | | | 0.95 | | 0.46-1.97 | | 0.90 | |
| Average | | 1.12 | | | | 0.75- 1.68 | | | 0.59 | | | | 1.08 | | 0.71-1.64 | | 0.72 | |
| Poor | | 1.00 | | | |  | | | - | | | |  | |  | |  | |
| Drinking water in dry season | |  | | | |  | | |  | | | |  | |  | |  | |
| Private tap | | 1.00 | | | |  | | |  | | | |  | |  | |  | |
| Protected spring | | 1.79 | | | | 0.48-6.62 | | | 0.39 | | | |  | |  | |  | |
| Public tap | | 1.06 | | | | 0.43-2.60 | | | 0.90 | | | |  | |  | |  | |
| Other | | 1.29 | | | | 0.82-2.02 | | | 0.27 | | | |  | |  | |  | |
| Drinking water rainy season |  |  | |  |  | | |  | |  | |  | | | |  | |  |
| Private tap | | | 1.00 | | | |  | | | |  | | | - |  | |  | |
| Protected spring | | | na | | | |  | | | |  | | | na |  | |  | |
| Public tap | | | 1.05 | | | | 0.45-2.47 | | | | 0.91 | | |  |  | |  | |
| Other | | | 1.38 | | | | 0.89-2.15 | | | | **0.15** | | | 1.23 | 0.51-2.98 | | 0.64 | |
| Water sufficiency for drinking and household chores | | | 0.99 | | | | 0.59-1.66 | | | | 0.97 | | |  |  | |  | |
| Frequency of washing drinking water container with soap | | |  | | | |  | | | |  | | |  |  | |  | |
| Never | | | 0.80 | | | | 0.34-1.91 | | | | 0.62 | | |  |  | |  | |
| Daily | | | 1.00 | | | |  | | | |  | | |  |  | |  | |
| Weekly | | | 0.95 | | | | 0.59-1.55 | | | | 0.85 | | |  |  | |  | |
| Container for fetching water | | |  | | | |  | | | |  | | |  |  | |  | |
| Clay pot | | | 0.57 | | | | 0.22-1.48 | | | | 0.25 | | |  |  | |  | |
| Plastic | | | 1.13 | | | | 0.73-1.76 | | | | 0.58 | | |  |  | |  | |
| Metal | | | 1.00 | | | |  | | | |  | | |  |  | |  | |
| Status of drinking water container | | |  | | | |  | | | |  | | |  |  | |  | |
| Covered | | | 1.00 | | | |  | | | |  | | |  |  | |  | |
| Uncovered | | | 0.74 | | | | 0.45-1.22 | | | | 0.24 | | |  |  | |  | |
| Drinking water container used for other activity | | | 1.35 | | | | 0.76-2.42 | | | | 0.31 | | |  |  | |  | |
| Water treatment prior to consumption | | | 0.89 | | | | 0.48-1.67 | | | | 0.72 | | |  |  | |  | |
| Water contamination with thermotolerant coliform | | | 1.09 | | | | 0.71-1.68 | | | | 0.68 | | |  |  | |  | |
| Sanitation in the household | | |  | | | |  | | | |  | | |  |  | |  | |
| No latrines | | | 0.53 | | | | 0.30-0.91 | | | | **0.02** | | | 0.52 | 0.29-0.92 | | **0.02** | |
| Water seal latrine | | | 1.00 | | | |  | | | |  | | |  |  | |  | |
| Open pit latrine with slab | | | 0.60 | | | | 0.32-1.13 | | | | **0.12** | | | 0.58 | 0.30-1.12 | | 0.11 | |
| Open pit latrine without slab | | | 0.64 | | | | 0.18-2.31 | | | | 0.50 | | |  |  | |  | |
| Soap for handwashing available | | | 1.48 | | | | 0.92-2.38 | | | | **0.10** | | | 1.53 | 0.93-2.52 | | 0.09 | |
| Waste disposal | | | 0.99 | | | | 0.66-1.48 | | | | 0.96 | | |  |  | |  | |
| Domestic animals | | |  | | | |  | | | |  | | |  |  | |  | |
| Possession of domestic animals | | | 1.22 | | | | 0.64-2.35 | | | | 0.55 | | |  |  | |  | |
| Animals held outside the house | | | 0.84 | | | | 0.54-1.32 | | | | 0.46 | | |  |  | |  | |
