# Supplementary material for: Intestinal parasite infections and associated risk factors among schoolchildren in Dolakha and Ramechhap districts, Nepal: a cross-sectional study
Source: Parasit Vectors. 2018 Sep 29;11:532. doi: 10.1186/s13071-018-3105-0 (PMC6162948; doi:10.1186/s13071-018-3105-0)
Supplement: Supplementary file 2 — Table S2. Results from univariate and multivariate logistic regression analyses for Giardia intestinalis. The multivariate global model includes a random intercept at the level of school adjusting sex, age, district where all the variables were assessed one by one and retained for the global model if their P-value is < 0.2. The final model was obtained by using backward selection with the same level of 0.2. (DOCX 36 kb) [file 13071_2018_3105_MOESM2_ESM.docx]

**Additional file 2: Table S2** Results from univariate and multivariate logistic regression analyses for *Giardia intestinalis*. The multivariate global model includes a random intercept at the level of school adjusting sex, age, district where all the variables were assessed one by one and retained for the global model if their *P*-value is < 0.2. The final model was obtained by using backward selection with the same level of 0.2.

| **Risk factor** | | ***Giardia lamblia* (n=181)** | | | | | | | | | | | |
| --- | --- | --- | --- | --- | --- | --- | --- | --- | --- | --- | --- | --- | --- |
|  |  | **Univariate analysis** | | | | | | | | | **Multivariate analysis** | | |
|  |  | **OR** | | | **95% CI** | | | ***P*** | | | **aOR** | **95% CI** | ***P*** |
| Sex | |  | | |  | | |  | | |  |  |  |
| Male | | 0.99 | | | 0.68-1.44 | | | 0.96 | | | 0.96 | 0.65-1.41 | 0.82 |
| Female | | 1.00 | | |  | | | - | | |  |  |  |
| Age | |  | | |  | | |  | | |  |  |  |
| 8-11 years | | 0.66 | | | 0.41-1.08 | | | **0.10** | | | 0.62 | 0.37-1.03 | 0.07 |
| >12 years | | 1.00 | | |  | | | - | | |  |  |  |
| District | |  | | |  | | |  | | |  |  |  |
| Dolakha | | 1.00 | | |  | | | - | | |  |  |  |
| Ramechhap | | 0.78 | | | 0.28-2.22 | | | 0.64 | | | 0.86 | 0.38-1.93 | 0.71 |
| Hygiene behavior | |  | | |  | | |  | | |  |  |  |
| Lower category | | 1.00 | | |  | | |  | | |  |  |  |
| Middle category | | 1.22 | | | 0.78-1.91 | | | 0.39 | | |  |  |  |
| Higher category | | 1.08 | | | 0.67-1.74 | | | 0.75 | | |  |  |  |
| Drinking water consumption | |  | | |  | | |  | | |  |  |  |
| From school | | 1.00 | | |  | | |  | | |  |  |  |
| From home | | 0.95 | | | 0.53-1.70 | | | 0.87 | | |  |  |  |
| Water risk behavior | |  | | |  | | |  | | |  |  |  |
| Playing (yes *vs* no) | | 0.97 | | | 0.64-1.46 | | | 0.88 | | |  |  |  |
| Fishing (yes *vs* no) | | 1.27 | | | 0.72-2.25 | | | 0.41 | | |  |  |  |
| Laundry (yes *vs* no) | | 1.10 | | | 0.72-1.68 | | | 0.66 | | |  |  |  |
| Domestic chores (yes *vs* no) | | 1.24 | | | 0.77- 2.00 | | | 0.37 | | |  |  |  |
| Sanitary practices | |  | | |  | | |  | | |  |  |  |
| Using latrine at school (yes *vs* no) | | 0.77 | | | 0.28-2.09 | | | 0.60 | | |  |  |  |
| Ethnicity of children | |  | | |  | | |  | | |  |  |  |
| Brahmin | | 1.18 | | | 0.67-2.08 | | | 0.57 | | | 1.18 | 0.65-2.13 | 0.58 |
| Chhetri | | 1.17 | | | 0.73-1.88 | | | 0.51 | | | 1.17 | 0.72-1.89 | 0.52 |
| Newar | | 0.92 | | | 0.35-2.36 | | | 0.86 | | | 0.99 | 0.38-2.59 | 0.99 |
| Tamang | | 1.00 | | |  | | |  | | | - |  |  |
| Janajati | | 6.47 | | | 0.87-48.26 | | | **0.07** | | | 7.94 | 0.96-65.44 | **0.05** |
| Caregiver`s education | |  | | |  | | |  | | |  |  |  |
| Never went school | | 1.00 | | |  | | |  | | | - |  |  |
| Primary education | | 0.77 | | | 0.45-1.30 | | | 0.33 | | | 0.82 | 0.47-1.41 | 0.47 |
| Secondary education | | 0.80 | | | 0.44-1.47 | | | 0.47 | | | 1.05 | 0.55-1.99 | 0.88 |
| Higher education | | 0.71 | | | 0.31-1.63 | | | 0.42 | | | 0.75 | 0.32-1.77 | 0.51 |
| Caregiver`s occupation | |  | | |  | | |  | | |  |  |  |
| Farmer | | 1.00 | | |  | | |  | | |  |  |  |
| Public services | | 0.71 | | | 0.31-1.63 | | | 0.42 | | | 0.67 | 0.28-1.61 | 0.37 |
| Business | | 0.51 | | | 0.21-1.26 | | | **0.14** | | | 0.47 | 0.18-1.21 | 0.12 |
| Other | | 0.32 | | | 0.11-0.95 | | | **0.04** | | | 0.29 | 0.10-0.88 | **0.03** |
| Socioeconomic status | |  | | |  | | |  | | |  |  |  |
| High | | 1.05 | | | 0.53-2.09 | | | 0.89 | | | 1.04 | 0.51-2.11 | 0.92 |
| Average | | 1.16 | | | 0.77-1.73 | | | 0.48 | | | 1.10 | 0.73-1.67 | 0.65 |
| Poor | | 1.00 | | |  | | |  | | |  |  |  |
| Drinking water in dry season | |  | | |  | | |  | | |  |  |  |
| Private tap | | 1.00 | | |  | | |  | | |  |  |  |
| Protected spring | | 2.37 | | | 0.58- 9.68 | | | 0.23 | | |  |  |  |
| Public tap | | 1.17 | | | 0.50-2.74 | | | 0.72 | | |  |  |  |
| Other | | 1.02 | | | 0.65-1.60 | | | 0.94 | | |  |  |  |
| Drinking water in rainy season |  |  |  |  | | |  | | |  |  |  |  |
| Private tap | | 1.00 | | | |  | | |  | |  |  |  |
| Protected spring | | na | | | |  | | |  | |  |  |  |
| Public tap | | 1.02 | | | | 0.44-2.34 | | | 0.96 | |  |  |  |
| Other | | 1.14 | | | | 0.73-1.76 | | | 0.57 | |  |  |  |
| Water sufficiency for drinking and household chores | | 1.05 | | | | 0.63-1.74 | | | 0.85 | |  |  |  |
| Frequency of washing drinking water container with soap | |  | | | |  | | |  | |  |  |  |
| Never | | 1.72 | | | | 0.77-3.83 | | | **0.18** | | 1.62 | 0.72-3.66 | 0.25 |
| Daily | | 1.00 | | | |  | | |  | | - |  |  |
| Weekly | | 1.39 | | | | 0.86-2.23 | | | **0.18** | | 1.36 | 0.84-2.20 | 0.21 |
| Container for fetching water | |  | | | |  | | |  | |  |  |  |
| Clay pot | | 0.54 | | | | 0.21-1.39 | | | 0.20 | |  |  |  |
| Plastic | | 1.14 | | | | 0.73-1.77 | | | 0.57 | |  |  |  |
| Metal | | 1.00 | | | |  | | |  | |  |  |  |
| Status of drinking water container | |  | | | |  | | |  | |  |  |  |
| Covered | | 1.00 | | | |  | | |  | |  |  |  |
| Uncovered | | 0.90 | | | | 0.54-1.48 | | | 0.68 | |  |  |  |
| Drinking water container used for other activity | | 1.67 | | | | 0.92-3.04 | | | **0.09** | | 1.43 | 0.77-2.65 | 0.26 |
| Water treatment prior to consumption | | 0.77 | | | | 0.41-1.47 | | | 0.44 | |  |  |  |
| Water contamination with thermotolerant coliform | | 0.93 | | | | 0.61-1.43 | | | 0.75 | |  |  |  |
| Sanitation in the household | |  | | | |  | | |  | |  |  |  |
| No latrines | | 0.65 | | | | 0.38-1.12 | | | **0.12** | | 0.66 | 0.38-1.13 | 0.13 |
| Water seal latrine | | 1.00 | | | |  | | |  | |  |  |  |
| Open pit latrine with slab | | 0.76 | | | | 0.41-1.42 | | | 0.39 | |  |  |  |
| Open pit latrine without slab | | 0.32 | | | | 0.07-1.57 | | | **0.16** | | 0.32 | 0.06-1.58 | 0.16 |
| Soap for handwashing available | | 1.33 | | | | 0.83-2.13 | | | 0.24 | |  |  |  |
| Waste disposal | | 1.05 | | | | 0.70-1.57 | | | 0.83 | |  |  |  |
| Domestic animals | |  | | | |  | | |  | |  |  |  |
| Possession of domestic animals | | 1.30 | | | | 0.69-2.46 | | | 0.42 | |  |  |  |
| Animals held outside the house | | 0.59 | | | | 0.38-0.92 | | | **0.02** | | 0.52 | 0.33-0.83 | 0.01 |
